# Mitochondria and Reactive Oxygen Species: Physiology and Pathophysiology

**DOI:** 10.3390/ijms14036306

**Published:** 2013-03-19

**Authors:** Subhashini Bolisetty, Edgar A. Jaimes

**Affiliations:** 1 Nephrology Division, University of Alabama at Birmingham, Birmingham, AL 35294, USA; E-Mail: subhashini@uab.edu; 2 Veterans Affairs Medical Center, Birmingham, AL 35233, USA

**Keywords:** mitochondria, reactive oxygen species, nitric oxide, hydrogen peroxide, mitochondria fission, mitochondria fusion, autophagy, mitochondria network, cell signaling

## Abstract

The air that we breathe contains nearly 21% oxygen, most of which is utilized by mitochondria during respiration. While we cannot live without it, it was perceived as a bane to aerobic organisms due to the generation of reactive oxygen and nitrogen metabolites by mitochondria and other cellular compartments. However, this dogma was challenged when these species were demonstrated to modulate cellular responses through altering signaling pathways. In fact, since this discovery of a dichotomous role of reactive species in immune function and signal transduction, research in this field grew at an exponential pace and the pursuit for mechanisms involved began. Due to a significant number of review articles present on the reactive species mediated cell death, we have focused on emerging novel pathways such as autophagy, signaling and maintenance of the mitochondrial network. Despite its role in several processes, increased reactive species generation has been associated with the origin and pathogenesis of a plethora of diseases. While it is tempting to speculate that anti-oxidant therapy would protect against these disorders, growing evidence suggests that this may not be true. This further supports our belief that these reactive species play a fundamental role in maintenance of cellular and tissue homeostasis.

## 1. Introduction

Mitochondria were first identified over a century ago and were initially termed as “bioblasts” by Richard Altmann who described them as “elementary organisms” living inside cells [1]. In fact this theory of endosymbiosis that mitochondria are the direct descendants of a bacterial endosymbiont is still one of the most widely accepted theories of mitochondrial evolution [2]. The term mitochondria, was later coined by Carl Benda and literally means “mitos-thread” and “chondrion-granule” [1]. The role of mitochondria in the cell was initially presumed to be only to generate energy in the form of adenosine triphosphate (ATP) and is still referred to as the “powerhouse of the cell”. However, research in the past few decades has provided compelling evidence to suggest that mitochondria are actively involved in a multitude of cellular activities including, signaling, proliferation and death. In fact, while most eukaryotic cells contain mitochondria, the size, number and location of mitochondria in a cell vary significantly based on the cellular needs. For instance, in neuronal cells, mitochondria accumulate predominantly at high energy demanding sites such as presynaptic terminals, nodes of Ranvier and active growth cones and branches [3]. Given the role of mitochondria in a variety of cellular processes, it is not surprising that damage to the mitochondria has been implicated in the pathogenesis of end-organ injury in a variety of diseases [4–23].

How does a single organelle control the fate of the cell? This question has captivated scientists and studies over the past few decades have revealed fascinating mechanisms. An extensively studied and established mechanism relies on the generation of free radical species that determine the outcome of the cellular processes involved. This review will mainly focus on the role of these reactive oxygen/nitrogen species in the modulation of cellular activities. We will summarize some of the major pathways and molecular mechanisms that are regulated by reactive oxygen species (ROS) and reactive nitrogen species (RNS) and given the wealth of knowledge that exists for these species, this review will highlight recent developments in ROS/RNS mediated signaling pathways and their role in the regulation of cellular processes, such as autophagy, mitochondria fusion and fission.

## 2. Mitochondria Structure and Function

**Figure 1:**
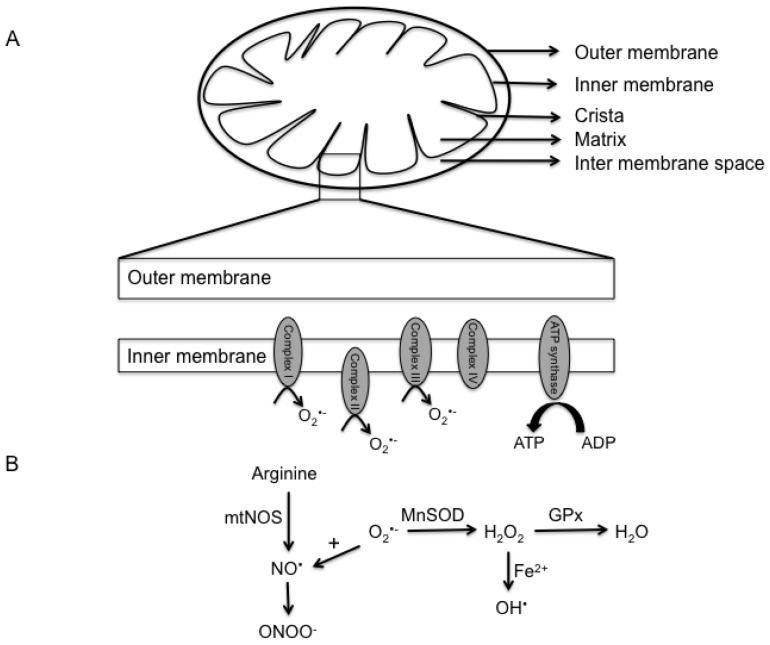
Mitochondria structure and generation of mitochondrial reactive species. (**A**) A schematic of a typical mitochondrion is represented. By virtue of its lipid bilayers, the mitochondrion can be subdivided into the outer membrane, inter-membrane space, inner membrane and matrix. The lower panel demonstrates the generation of superoxide anion through the different complexes of the electron transport chain; (**B**) Amplification of the free radical cycle. Superoxide generated during the electron transport chain can react with nitric oxide to form peroxy nitrite species. Alternatively, superoxide is converted by manganese superoxide dismutase to hydrogen peroxide, which is subsequently converted to water by glutathione peroxidase. In the presence of iron, hydrogen peroxide is rapidly converted to the highly reactive hydroxyl ion.

Mitochondria are unique organelles as its structure provides compartmentalization of metabolism (Figure 1A). They are very complex organelles that contain two phospholipid bilayers, by virtue of which they can be categorized into 4 different segments: the outer membrane, inter-membrane space, inner membrane and matrix [24–26].

The outer membrane of the organelle is identical to the plasma membrane in its content (equal ratio of protein to phospholipid content by weight). It contains porins that allow molecules that are less than 5 KDa to freely diffuse through. However, larger proteins require the presence of a mitochondria targeted sequence that will enable binding to specific transporters (translocase of the outer membrane—TOM and inner membrane—TIM) on the membrane for entry into the organelle [26–29]. The outer membrane therefore mainly serves as a permeability barrier to the cytosolic components. Until recently, it was presumed that the inter membrane space had no specific function and was identical to the cytosol in its contents. However, emerging studies have suggested an important role for this space in maintaining mitochondrial homeostasis, including protein sorting and lipid homeostasis (reviewed in [30]). The inner membrane of the mitochondria is perhaps the single most extensively studied cell membrane component due to its relative importance in oxidative phosphorylation. This membrane comprises of the highest number of proteins per phospholipid moiety in a cell. These proteins are integral to the electron transport chain, ATP synthesis and transport [31,32]. The inner membrane is also distinct from other membranes by the presence of cristae (invaginations of the membrane), which allow for compartmentalization and increases the surface area. The inner membrane is also less permeable to ions and molecules and helps in compartmentalization through separation of the mitochondrial matrix from the cytosolic environment, thereby acting as an electrical insulator and chemical barrier [32]. This helps in maintenance of the electron gradient across the membrane, which enables generation of ATP. The mitochondrial matrix of mammalian cells contains the mitochondrial DNA (16.5 kilobase genome) that encodes for nearly 13 proteins, some of which are involved in oxidative phosphorylation. The remaining proteins required for the normal function of the mitochondria are encoded by the nuclear genome and imported into the mitochondria [33]. The matrix also contains a majority of the enzymes required for the citric acid cycle, which oxidizes acetyl coenzyme A and in the process generates energy in the form of nicotinamide adenine dinucleotide (NADH) and flavin adenine dinucleotide (FADH_2_). These molecules then serve as substrates for oxidative phosphorylation by the proteins in the inner membrane to generate cellular energy in the form of ATP.

## 3. Reactive Oxygen and Nitrogen Species

Halliwell and colleagues described free radicals as “any species capable of independent existence that contains one or more unpaired electrons” [34]. The term ROS simply refers to a variety of reactive molecules that are derived from oxygen and can be free radicals (superoxide (O_2_^•−^) or hydroxyl radical (OH^•^)) or non-radicals (hydrogen peroxide (H_2_O_2_)). Similarly, they can be further classified as ions (O_2_^•−^) or non-ions (H_2_O_2_). On the other hand, RNS refers to reactive species derived from nitrogen and can be broadly classified as ions (peroxynitrite (ONOO^−^)) or non-ions (Nitric Oxide (NO^•^)). These reactive species are formed at low levels during the execution of physiological functions of the cell. In fact, the electron transport chain is responsible for most of the superoxide that is generated through partial reduction of oxygen (Figure 1A) and is reviewed in detail in the following sections. Furthermore, they are formed at different rates in a cell and differ in their activity. In terms of activity, hydroxyl radical is the most reactive species known and is by in large responsible for the cytotoxic effects of ROS. In contrast, reactive species such as nitric oxide and hydrogen peroxide are less reactive and have shown to play an important role in several cellular activities. This is discussed in detail in the following sections.

Other enzymatic systems responsible for the generation of these reactive species include but are not limited to respiratory burst enzymes (such as NADPH oxidases –Nox1-5), amino acid oxidases, cytochrome P450 enzymes, cycloxygenases, lipoxygenases, xanthine oxidase [35–41]. Addressing each of these systems is beyond the scope of this review and hence we will focus specifically on mitochondria associated reactive species.

As discussed above, mitochondria are one of the most active organelles in the cell, consuming nearly 90% of the total oxygen content in the cell to enable oxidative phosphorylation and ATP synthesis [42]. Given that low levels of superoxide are constantly generated during normal respiration by healthy mitochondria, several pathways evolved to detoxify this anion. For example, manganese superoxide dismutase (MnSOD) is a mitochondrial matrix enzyme that rapidly converts superoxide to hydrogen peroxide, another reactive species. This molecule can then be converted to water by catalase or glutathione peroxidase in the mitochondria or following diffusion into the cytosol. In addition to these enzymes, cells are equipped with a variety of antioxidant molecules, such as glutathione, ascorbic acid and α-tocopherol, which are capable or reducing ROS. Gluthathione contains a sulphahydryl group that is oxidized by ROS. Therefore, glutathione protects the vital mitochondrial components from being targeted by ROS by serving as a substrate. However, damaged and dysregulated mitochondria generate excessive amounts of superoxide which can damage several mitochondrial components, including proteins, lipids and DNA. These reactions then lead to a vicious cycle of further generation of reactive species and ultimately cell death. While hydrogen peroxide is a reactive molecule, in the presence of transition metals, such as iron, it can be converted to hydroxyl ion via the Fenton reaction. This iron is thought to be released through destabilization of ferritin and other iron containing proteins by superoxide anion [43–46]. Alternatively, superoxide may react with nitric oxide to generate peroxynitrite species. The details of these reactions are presented in Figure 1B.

Over the past few decades, research on these reactive species has soared in both physiology and pathology. With the advent of novel technology, researchers have now been able to shed light on the source of reactive species generation in the different sub-compartments of the mitochondria which has been extensively reviewed by Lenoz [47]. To summarize, it was demonstrated that complex I, also referred to as NADH CoQ reductase, catalyzes the transfer of electrons from NADH to coenzyme Q, which is accompanied by translocation of protons from the matrix to the intermembrane space. There is now evidence to suggest that complex I is involved in ROS production, specifically superoxide [48–50]. Similarly, succinate dehydrogenase, complex II enzyme is responsible for the reduction of CoQ and has also shown to be involved in generating low levels of superoxide anion [48,51]. Complex III (ubiquinol cytochrome c reductase), on the other hand has shown to be responsible for the superoxide generation in the intermembrane space. Superoxide generation by this complex is significantly enhanced when the electron transfer is reduced, either due to inhibition in respiration (actinamycin A) or an increase in membrane potential [52,53]. Interestingly, the contribution of each of these enzymes to ROS production is different in different tissues and during disease conditions. For instance, while complex III has been implicated as the major source of superoxide in the heart, complex I seem to be of prime importance in the brain [54–57]. Additionally, enzymes such as Glycerol-3-phosphate dehydrogenase, Monoamine oxidase, Dihydrolipoamide dehydrogenase and Electron-transferring-flavoprotein dehydrogenase have also been implicated in ROS production [58–63].

As evident from the reactions described above, most of the superoxide generated is either in the matrix or on the inner membrane of the mitochondria that faces the matrix. While most of the reactive species generated within the mitochondria is superoxide anion, MnSOD rapidly converts it to hydrogen peroxide. Although hydrogen peroxide is more stable than superoxide, it can freely diffuse out of the mitochondria into the cytosol, thereby reducing the harmful effects of these reactive species to the mitochondria. It has also been suggested that in the presence of excessive superoxide, MnSOD is oxidized and this further compromises the antioxidant capacity of the mitochondria and enhances oxidative stress [64]. Alternatively, superoxide may be carried to the cytoplasm by voltage-dependent anion channels [65].

Nitric oxide (NO^•^) is another reactive species that is generated by the mitochondria and research on this molecule has gained momentum over the past few decades. Nitric oxide is generated during the breakdown of arginine to citrulline by a family of NADPH-dependent enzymes called nitric oxide synthases (NOS). The importance of this enzyme in physiology is underscored by the fact there are several isoforms of NOS, including an endothelial constitutive isoform (NOS3), an inducible isoform that is expressed in several cell types (NOS2) in response to pro-inflammatory stimuli and produces large amounts of NO^•^ and a neuronal isoform (NOS1). Recently, a mitochondrial isoform of NOS referred to as mtNOS has been described [66]. This isoform was found to be responsive to changes in calcium concentration in the matrix and to play an important role in modulating mitochondrial respiration. Following this initial study, several groups have identified the presence of mtNOS in the mitochondria of cells from different tissues including liver, brain and kidney [67–72]. However, and in spite of these studies there is still debate on the existence of this isoform and indeed some studies suggest that the presence of NO^•^ in the mitochondria may reflect pathways independent of NOS activity that may include nitrite reductase activity and the electron transport chain [65,72–75]. Once generated, NO^•^ can inhibit respiration by binding to heme groups in the proteins of the electron transport chain, including cytochrome c oxidase [76–81]. Irrespective of the source of NO^•^, its presence in the mitochondria can alter the activity of a number of processes including respiration, mitochondrial biogenesis and oxidative stress through increased production of reactive oxygen and nitrogen species and thereby impact cell physiology [82–94].

As described above, mitochondria are a major source of reactive oxygen and nitrogen species. Interestingly, these species in turn regulate the mitochondrial activity through several mechanisms, including mitochondrial biogenesis, mtDNA damage, lipid peroxidation and mitochondrial membrane permeability transition (reviewed in [83,86,89–98]). The physiological and pathological role of these reactive species in cellular activities will be discussed below.

## 4. Autophagy

**Figure 2:**
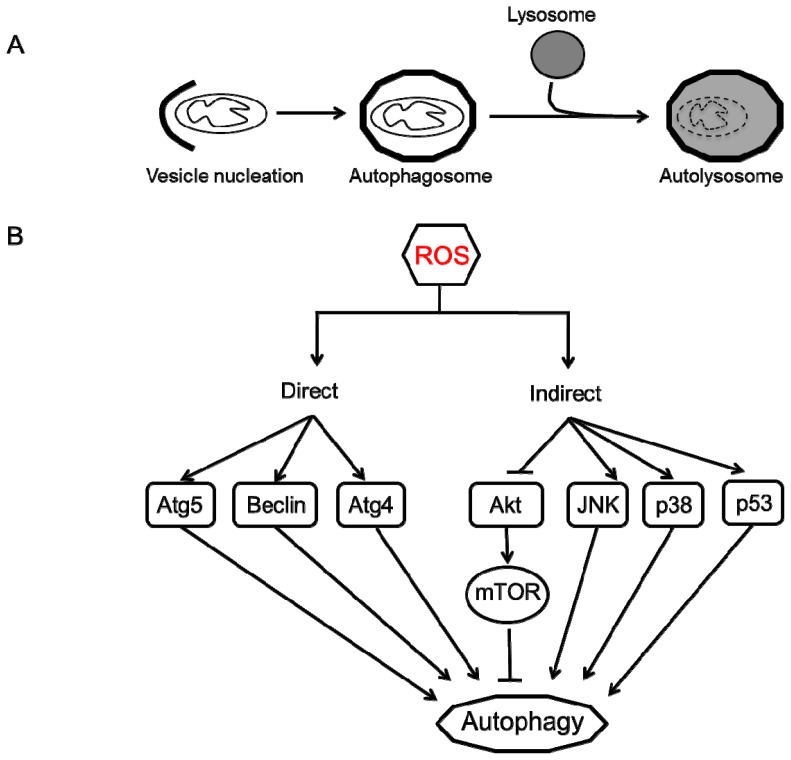
Autophagy process and regulation by ROS. (**A**) Autophagy begins with vesicle nucleation where the damaged organelles (mitochondria) are sequestered to form an autophagosome. This vesicle fuses with the lysosome to form an autolysosome where the contents are degraded by lysosomal hydrolases and nutrients are recycled to the cytoplasm; (**B**) ROS can regulate autophagy in two ways: direct and indirect. Direct regulation involves modification of key proteins involved in the autophagy process including Atg4, Atg5 and Beclin. Indirect regulation by ROS involves alteration of signaling pathways such as JNK, p38 that can induce autophagy. On the other hand, ROS may inhibit Akt signaling and downstream mTOR and thereby induce autophagy.

Autophagy (meaning self-eating) is an evolutionarily conserved catabolic process that involves an intracellular degradation system in which cytoplasmic components, such as organelles, protein aggregates and other macromolecules are directed to the lysosome through a physiologically regulated process to maintain cell homeostasis. While three different types of autophagy (micro-, macro- and chaperone-mediated autophagy) have been described, the most studied process in mammalian cells is macro-autophagy. As the name suggests, it involves degradation of large moieties such as organelles and protein aggregates through a tightly regulated process. This process begins with the isolation and sequestration of the cytoplasmic components by a double-layered lipid membrane that forms an autophagosome. This vesicle then fuses with a lysosome to form an autolysosomes where the sequestered cellular components are degraded by the lysosomal enzymes (Figure 2A) [99–102]. Although this process may appear to be self-destructive, it is an extremely efficient recycling strategy that is vital to maintain cell homeostasis and generates amino acids, fatty acids and energy (ATP) that are used for macromolecular synthesis. There have been at least 35 Atg (**A**u**t**opha**g**y) related genes identified in yeast whereas their mammalian orthologs have not been completely characterized [103,104].

Autophagy has been associated with a number of diseases, including but not limited to cancer (breast, ovarian, prostate and colon), neurodegenerative diseases (Alzheimer’s, Parkinson’s and Huntington’s disease), myodegenerative diseases (muscular dystrophy, X-linked myopathy), Crohn’s disease, diabetes and several other inherited diseases such as Danon disease, Pompe disease. However, the role of autophagy in protection or disease progression has remained a conundrum [101,105–116]. Nevertheless, research in the past decade has unraveled the autophagy process and provided compelling evidence to suggest a protective role for regulated and controlled autophagy [106,107]. Nutrient-starvation ROS generation is one of the key modulators of autophagy in normal and cancerous cells. In a comprehensive study, Chen and colleagues provide irrefutable evidence to suggest that mitochondrial ROS are important mediators of autophagy and autophagic cell death in transformed cells and cancer cells [117]. They demonstrate that overexpression of SOD or use of ROS scavengers are capable of lowering autophagy and cell death in the presence of electron transport chain inhibitors, rotenone and thenoyltrifluoroacetone [117]. Mitochondrial ROS can regulate autophagy in two major pathways: by direct modification of the autophagy proteins or by altering the proteins that are indirectly involved in the autophagy process.

ROS, specifically, hydrogen peroxide that is generated during starvation, modulate the activity of Atg4, an essential cysteine protease in the autophagic pathway, through a series of redox reactions. Atg4 cleaves the c-terminus of Atg8, enabling the addition of phosphatidylethanolamine (PE) to Atg8 and subsequent conjugation of this protein on the autophagosomal membrane, leading to autophagosome maturation. However, Atg8-PE also serves as a substrate for Atg4, which allows for efficient recycling of Atg8. This protease is therefore tightly regulated and research now points to hydrogen peroxide as a mediator of this redox signaling. Hydrogen peroxide oxidizes Atg4 following the initial cleavage, thereby allowing the autophagosome completion. Once the lysosome fuses with this vesicle, Atg4 is re-activated and recycles Atg8 for another cycle of autophagy. This is the first description of ROS as a signaling molecule that triggers autophagy as a cell survival mechanism [107]. While the mechanism is not clearly understood, several studies have demonstrated an upregulation of beclin 1, a protein involved in autophagy initiation, in the presence of ROS [118–121]. However, it is still unknown whether this cysteine rich protein is also modulated by redox activity.

With regard to ROS-mediated indirect regulation of autophagy, hydrogen peroxide and superoxide anion can modulate the activity of a number of signaling pathways that induce autophagy. Using glioma cells as a model to unravel autophagic mechanism in cancerous cells, several groups have identified key regulatory effectors of ROS that play a major role in the autophagy pathway. One such effector is the mammalian target of rapamycin (mTOR) that is actively involved in a variety of cellular processes including transcription, proliferation, motility and survival. Hydrogen peroxide can disrupt the mitochondrial membrane potential, leading to an inhibition in Akt/mTOR signaling pathway that is capable of inducing autophagy [122,123]. Similarly, several studies have demonstrated the role of ROS in regulation of MAPK (mitogen activated protein kinase) pathways. Increased ROS (hydrogen peroxide and nitric oxide) levels in cardiomyocytes or skeletal muscle, induces autophagy that is dependent on p38 signaling [115,124]. Additionally, using a number of different tumor cell lines, Wong and colleagues demonstrated that ROS and downstream activation of ERK and JNK pathways were responsible for autophagy induction [125]. In another study, ROS mediated glycogen synthase kinase-3 activity was responsible for cadmium induced autophagy in mesangial cells [126]. Of note, even non-mitochondria associated NADPH oxidase–generated ROS can induce autophagy, implying that irrespective of the source, ROS can act as signaling molecules. This phenomenon has been observed in several immune cells (macrophages and neutrophils) that induce ROS-mediated autophagy to enable destruction of phagocytosed microbes [127–130].

While the majority of the research discussed above demonstrates a role for ROS in inducing autophagy, there is strong evidence to suggest that autophagy may in turn regulate mitochondrial network by eliminating damaged mitochondria. Kissova and colleagues were the first to demonstrate in yeast that an outer mitochondrial protein, Uth1p was responsible for the early selective degradation of mitochondria by autophagy during stress induced by nutrient deprivation or rapamycin [131]. This phenomenon gave rise to “mitophagy”, a term coined by Leimaster to describe a sub-type of macroautophagy where damaged mitochondria were sequestered by autophagosomes and removed to maintain mitochondrial homeostasis [132]. Since this discovery, the molecular mechanisms regulating mitophagy in yeast have been identified. However, in the mammalian system, research has been mainly focused on parkin/pink1 (PTEN-induced putative kinase protein 1) mechanism. PINK1 is a mitochondrial kinase that may serve as a guardian to the mitochondria through its ability to identify depolarized mitochondria. Under normal conditions, PINK1 undergoes voltage-dependent lysis and is removed from the mitochondria. However, in damaged mitochondria that have low membrane potential, PINK1 accumulates on the mitochondrial surface and recruits parkin [133,134]. Parkin ubiquitinates mitochondrial proteins, including voltage-dependent anion channel 1 (VDAC1), and recruits autophagic machinery to the damaged mitochondria for removal [133,135–138]. Recently, Egan and colleagues demonstrated that mammalian ortholog of Atg1, ULK-1 is required for mitophagy. In the absence of this protein, there was a decrease in starvation-induced mitophagy and an associated increase in the number of aberrant mitochondria in mouse embryonic fibroblasts and hepatocytes, suggesting that ULK-1 is a key regulator of mitochondrial homeostasis [139].

In summary, these studies suggest that autophagy is a complex process that is regulated by ROS in multiple ways (Figure 2B). Autophagy is a double-edged sword, with both cytoprotective and cytotoxic capabilities. Whether it inhibits cell death by removing damaged organelles (mitochondria) or induces cell death depends on many factors, including ROS. Mild ROS induces autophagy (or mitophagy) as a survival mechanism to eliminate damaged mitochondria that are responsible for uncontrolled ROS generation. On the contrary, high levels of ROS or sustained exposure to ROS can alter signaling pathways that converge to induce autophagic or apoptotic cell death [116,140].

## 5. Mitochondria Network

Mitochondria are complex organelles that exist as a tubular network in cells. Two opposing pathways that must exist in equilibrium sustain this dynamic filamentous network: mitochondria fusion and fission. While the molecular mechanism and signaling involved in these two processes is only now being understood, it is well established that dysregulation of these pathways can lead to cellular dysfunction. Mitochondria fusion, mediated by mitofusin-1 and mitofusin-2 are responsible for the lengthening and tethering of adjacent mitochondria to form a network. On the contrary, mitochondria fission involves division of mitochondria and is mediated by fission-1 and dynamin-related protein 1 (Drp-1). Under healthy conditions, the mitochondrial tubular network is established through increased fusion. However, during oxidative stress, mitochondria fission prevails and the filamentous network is broken down to fragmented mitochondrial puncta [141,142].

Hydrogen peroxide, a major contributor to oxidative damage in cells, was shown to induce mitochondria fission in a variety of cells, including fibroblasts. This fragmentation has been shown to be dose- and time-dependent and reversible, suggesting that mitochondria dynamics may be involved in signaling during cellular stress [143–145].

**Figure 3:**
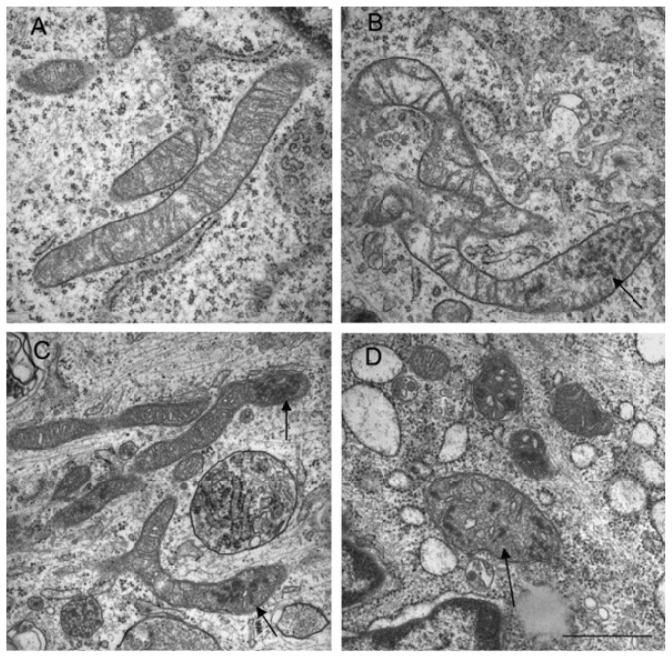

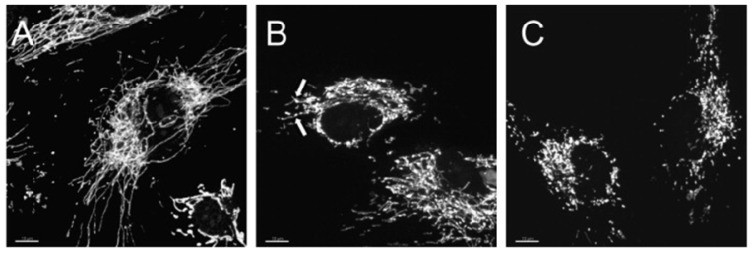
Changes in mitochondrial structure following hydrogen peroxide treatment. (Top panels) Transmission electron micrographs of mitochondria in untreated (**A**) and hydrogen peroxide treated cells (**B**,**C**,**D**). Electron dense granules (arrows) and fragmented mitochondria were observed. Bar = 1 μm. (Bottom panels) Mitochondria were stained with Mitotracker Red and existed as tubular (**A**), intermediate (**B**) (tubular with swollen regions) and fragmented (**C**). Bar = 10 μm.

**Figure 4:**
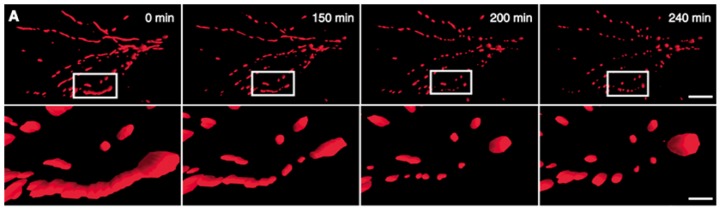
NO^•^ triggers mitochondrial fission. (**A**) 3D time-lapse microscopy of mitochondria undergoing fission in a dendritic arbor of a neuron. Neurons were transfected with Mito-DsRed2, pretreated with the pan-caspase inhibitor zVAD-fmk methyl ester (100 M), and exposed to SNOC (200 M). Images were 3D iso-surface rendered. Frames depict representative time points of the movie demonstrating mitochondrial fragmentation within 3 h of NO^•^ exposure (upper panels; scale bar, 15 μm) and closeup views (lower panels; scale bar = 3 μm).

In a recent study, Makino and colleagues demonstrated that superoxide anion was able to induce mitochondrial fragmentation in coronary endothelial cells isolated from a diabetic mouse. Supplementation with TEMPOL, a superoxide scavenger was able to inhibit mitochondrial fragmentation, demonstrating a causal role for superoxide in this process [146]. This was also corroborated by another study that treated HUVEC cells with hydrogen peroxide and demonstrate mitochondria fragmentation, which was suppressed in the presence of an anti-oxidant, *N*-acetylcysteine (Figure 3) [145]. In another study using neuronal cells, nitric oxide had a profound fission effect that was rescued by antioxidants (Figure 4) [147,148]. Furthermore, mitochondrial fission in neurons occurs prior to the onset of neuronal loss in an animal model of stroke [147]. This was further demonstrated in the kidney following ischemia reperfusion and cisplatin nephrotoxicity [149,150]. Conversely, Yu *et al.* demonstrated that mitochondria fission plays an important role in ROS overproduction and demonstrate that inhibition of fission reduces ROS generation by the mitochondria [151]. While all these studies implied a role for ROS/RNS in mitochondria fission, a recent study by Giedt and colleagues demonstrated that during simulated ischemia reperfusion in endothelial cells, DRP-1 activation (phosphorylation and oligomerization) was enhanced. This was inhibited by *N*-acetylcysteine or l-NG-Nitroarginine Methyl Ester (l-NAME, a NOS inhibitor), suggesting that oxidative/nitrosative stress drives Drp1 activation and translocation to mitochondria, which could be the underlying mechanism that induces mitochondrial fission in these studies [152,153]. These studies suggest that different species of ROS can initiate or act as a messenger in the signaling process leading to mitochondrial fragmentation and when persistent, it can lead to chronic mitochondria fission and apoptosis.

While a majority of these studies point to the role of ROS/RNS to activating fission, there is also evidence suggesting that inhibition of fission may be detrimental to the mitochondria network and cellular homeostasis. Several studies have demonstrated that inhibition of fission led to a decrease in cytochrome c release and delayed apoptosis [154–158]. Similarly, proteins involved in mitochondria fusion have shown to play an anti-apoptotic role [157,159]. However, recent evidence in a number of models suggests that inhibition of fission can lead to mitochondrial dysfunction, increase in ROS levels accompanied by loss of mtDNA and concomitant reduction in energy production and autophagy [160,161].

It is now evident from a variety of studies that ROS affects mitochondrial dynamics and leads to fragmentation of mitochondria tubules. It is also apparent that the mitochondria network plays an important role in mitochondrial function, including respiration, ATP production, apoptosis and functional complementation of mitochondrial DNA mutations and/or damaged proteins. However, it has been suggested that transient or low levels of ROS may in fact benefit the network by selectively removing the dysfunctional mitochondria through autophagy. This idea was proposed using a simulation model that suggested that the vulnerability of the mitochondria network to the harmful effects of ROS is dependent on the dynamics of the network. As one might expect, the healthy mitochondria in close association with the ROS producing dysfunctional mitochondria would be the first to be affected, starting a vicious cycle of uncontrolled and amplified ROS generation. As a result, if these damaged mitochondria were not isolated by fission, they would contaminate the entire network leading to a decrease in energy production and cell proliferation ultimately leading to cell death [162–164].

Nevertheless, it is now clear that defects in mitochondrial fusion or fission alter the susceptibility of cells to undergo apoptosis as documented in a variety of disorders, including heart failure, ischemia reperfusion injury, diabetes, Parkinson’s disease, muscle atrophy, Alzheimer’s disease and aging [141,149,165–172]. Most importantly, these studies provide mechanistic evidence for a role for ROS/RNS and redox signaling in contributing to perturbations in the mitochondrial dynamics and suggest the potential of mitochondria-targeted therapeutics in diseases that involve mitochondrial fragmentation due to uncontrolled ROS/RNS generation.

## 6. Signaling

Until recently, ROS generation was considered a bane to all aerobic organisms. In fact, this theory was sustained by research that continued to demonstrate generation of high levels of superoxide and nitric oxide by phagocytic cells and its role in host defense [173,174]. In stark contrast, non-immunity based research provided evidence to suggest that these molecules (nitric oxide and hydrogen peroxide) were generated in low levels in other cells, suggesting a distinctive role from its phagocytic activity. In fact, more than three decades ago, pioneers in nitric oxide biology such as Moncada, Ignarro and Furchgott demonstrated a vasodilatory property for this molecule, a discovery that led to their Nobel Prize in 1998 [175–177]. Following this observation, several investigators have established the importance of nitric oxide and other reactive oxygen intermediates in vascular homeostasis [178–187]. Indeed, there is compelling evidence today that demonstrates that these reactive species function as a second messenger in signal transduction and affect cellular function in different tissues [188–205]. Here in we will only focus on the pathways that best illustrate the role of these species in modulating signal transduction.

Since the discovery of a dichotomous role of reactive species in immune function and signal transduction, research in this field grew at an exponential pace and the pursuit for the mechanisms involved began. In a simplistic manner, these regulations can be hypothesized to occur through simple oxidative/nitrosative reactions. For instance, cysteine residues on proteins may be oxidized and hence significantly alter the activity of these proteins. These proteins (e.g., transcription factors, kinases, phosphatases) may then affect downstream signaling cascades that affect cellular responses to stimuli. Alternatively, these residues may be modified through nitrosylation by reactive nitrogen species. More importantly, this redox mechanism may occur at multiple levels in the signal transduction cascade and thereby ultimately alter the fate of a cell.

Earlier research was primarily focused on the ability of hydrogen peroxide to mediate cellular signaling due to its relative stability and ease of measurement. Hydrogen peroxide can also be converted to the highly reactive hydroxyl ion in the presence of a transition metal, such as iron, to amplify oxidative stress and activate cell death pathways. However, there is some consensus that the level of ROS itself may dictate the fate of the cell by modulating different redox-sensitive transcription factors and hence lead to diverse biological responses. For instance, low ROS levels induce Nuclear factor erythroid 2-related factor 2 (Nrf2), a potent transcription factor responsible for the induction of several antioxidant enzymes, including but not limited to NADPH quinone oxidoreductase (NQO1), glutathione S-transferase, heme oxygenase-1 (HO-1), ferritin and γ-glutamylcysteine synthetase [206,207]. When there is a moderate increase in ROS levels, NF-κB and AP-1 are activated and when there are extremely high levels or persistent ROS accumulation in the cell, membrane permeability transition pore is opened, cytochrome c is released from the mitochondria and apoptosis is triggered. These signaling pathways are regulated in multiple ways, some of which are independent of ROS.

## 7. Modulation of Nrf2 by Reactive Oxygen and Nitrogen Species

Under normal conditions, low levels of ROS generated by the mitochondria are neutralized or scavenged by anti-oxidants and scavengers present in the cell, many of which under the regulation of Nrf2. During mild increases in ROS, Nrf2 translocates to the nucleus, binds to the anti-oxidant response element (ARE) present on stress responsive genes and activates the promoters [207]. It has been suggested that more than 200 genes involved in the cellular antioxidant and anti-inflammatory defense were regulated by Nrf2 (reviewed in [208–214]) and suggesting that the activation of Nrf2 is tightly regulated. Indeed, the regulation of Nrf2 is complex and involves multiple factors including phosphorylation, protein interaction and stability [215–217]. Nrf2 stability is the most widely studied and involves a zinc zinc metalloprotein, Kelch-like ECH associated protein1 (Keap1). Under unstimulated conditions, Nrf2 is sequestered in the cytosol by Keap1 and is targeted for ubiquitin-dependent proteosomal degradation [218–221]. A variety of stimuli can induce Nrf2 by disrupting the Keap1-Nrf2 interaction and inhibiting Nrf2 ubiquitination (reviewed in [215,222–228]. With regard to hydrogen peroxide and nitric oxide, both have shown to oxidize several cysteine residues on Keap1, thereby forming intra- and intermolecular disulphides and inactivating Keap1 [229–234]. Interestingly, the majority of Nrf2 activators increase reactive oxygen and nitrogen species. It is therefore tempting to speculate that Nrf2 activation necessitates the presence of reactive species. While Nrf2 activation and its downstream signaling are important mediators of anti-oxidant signaling during exposure to low levels of reactive species, it is widely believed that NF-κB and AP-1 signaling pathways are switched on to protect against increased cellular stress.

## 8. NFκB Activation by Reactive Species

Another interesting signaling pathway that is modulated by reactive species is NFκB. Since its discovery by Baltimore almost three decades ago [235], it has been implicated in the regulation of various cellular responses to stress such as apoptosis and inflammation [236–251]. Generation of various NFκB transgenic knockout mice further established its role in these processes [252]. Although it was initially presumed that these factors were expressed only in B lymphocytes, growing evidence indicates its presence in most mammalian cells. Furthermore, aberrant NFκB signaling has been associated with the pathogenesis of a number of diseases, including cancer, atherosclerosis and schizophrenia [253–256]. NFκB signaling is one of the most complex pathways and consists of five different transcription factors known as p65, p50, p52, c-Rel ad RelB. These factors contain a homology domain in their N-terminus (Rel homology domain, RHD) that enables interaction with DNA and dimerization with other RHD containing factors. NFκB is predominantly regulated by a family of inhibitory proteins, IκB that sequester this protein in the cytoplasm using this domain [236,257,258]. The IκB family consists of seven IκB proteins that can modulate NFκB signaling. The contributory role of the NFκB family to transcriptional regulation of genes is complex and continues to grow. For instance, while p50 and p52 homodimers have inhibitory effect on transcription on numerous genes, heterodimers of these factors stimulate transcription [259–261]. NFκB is induced by a variety of stimuli, including cytokines and oxidative stress and plays an important role in a variety of processes such as cell proliferation, inflammation and apoptosis. It achieves this versatility through its regulation of a multitude of genes, including those involved in reactive species generation such as NOS and cyclooxgenase 2 [262–268]. Herein we will highlight the role of reactive species in regulating NFκB signaling.

NFκB was one of the first transcription factors to be described as an oxidative stress responsive transcription factor. This was first demonstrated by Schreck and colleagues in T cells using hydrogen peroxide [269] and further confirmed by incubating cells with scavengers of ROS and demonstrating that ROS-mediated NFκB activation was inhibited [269,270]. Later studies demonstrated that hydrogen peroxide and nitric oxide could activate NFκB signaling in a variety of cells, including cancer cell lines (MCF-7, HeLa, LNCaP), fibroblasts, chondrocytes, lymphocytes, macrophages, epithelial and endothelial cells [269,271–281]. Similarly, NFκB activation was impaired in the presence of anti-oxidants, such as NAC, MnSOD and glutathione peroxidase [274,282–285]. On the other hand, high levels of nitric oxide have shown to inhibit NFκB activation in endothelial cells, hepatocytes, cancerous cells, macrophages and T cells [286–292].

While activation of NFκB signaling promotes survival during stress mediated by a variety of insults, it has also shown to induce death (reviewed in [293]). It seems plausible that while ROS activates NFκB, NFκB signaling may in turn inhibit ROS production to promote survival. The list of anti-oxidant genes induced by NFκB is extensive and comprises of MnSOD, Ferritin, catalase, glutathione s-transferase, heme oxygenase-1, glutathione peroxidase and many others. On the contrary, NFκB plays an important role in inflammation through upregulation of ROS producing enzymes such as NADPH oxidase, NOS, xanthine oxidase, cyclooxygenases. Interestingly, another mechanism by which reactive species may affect signaling is through its oxidation potential. While on one hand, oxidative stress increases NFκB activity, higher levels of ROS may lead to oxidation of NFκB and reduce its activity. For instance, transcriptional activation of NOS requires NFκB [288,294], and once NO^•^ is formed, it can either activate or inhibit NFκB signaling [272,289,294–299].

It now seems more certain that reducing conditions are required in the nucleus for NF-κB DNA binding [300,301], whereas oxidizing conditions in the cytoplasm promote NF-κB activation [275,302,303]. It has been suggested that reactive species stimulate NFκB in the cytosol whereas inhibit its activity in the nucleus [304]. The DNA binding ability of this transcription factor has shown to be modulated by redox status in the cell [300,305,306]. There is evidence to suggest that redox factor protein, Ref-1 reduces cysteine 62 in NFκB in the nucleus and this reaction is required for NFκB to bind to DNA [305]. Conversely, oxidation of this residue inhibits binding to DNA [306]. In addition, glutathionylation of NFκB in the presence of reactive oxygen species led to a decrease in its DNA binding ability and downstream transcriptional activity [307].

Another mechanism by which NFκB can be modulated is through phosphorylation of NFκB or its inhibitor, IκB. Hydrogen peroxide has shown to alter the activity of IκK, a kinase that phosphorylates IκB and hence allows NFκB translocation to the nucleus [308]. Alternatively, ROS can increase the activity of IκK indirectly through modulation of Akt signaling [309]. Furthermore, it was recently demonstrated that ROS mediated phosphorylation of RelA on serine 276 is essential for TNF-α induced NFκB activation and signaling [310]. To summarize, the regulation of NFκB by reactive species and the inverse is extremely complex and more research is warranted for a complete understanding of the mechanisms involved.

## 9. Other Signaling Pathways

Due to the vast wealth of knowledge available on ROS and RNS signaling, we only focused on two of the most important pathways. However, these species have been implicated in MAPK [311–315], HIF-1 α [316,317], p53 [318–323], AP-1 [313,324,325], SP-1 [313,326,327], apoptosis (caspase regulation) [328–332], cytokine [333–337] and fibroblast-derived growth factor [338–340] and platelet derived growth factor [97,340–344]. Of note, this list is not all encompassing and is constantly growing as research in this field progresses. Furthermore, ROS and RNS mediate mitochondrial cell death signaling pathways and have been extensively reviewed and hence not discussed in this review [345–353].

## 10. Antioxidants

As discussed in the previous sections, while hydrogen peroxide and NO^•^ are important mediators of cellular processes, higher levels of these species or the presence of highly reactive species such as hydroxyl radical and peroxynitrite can potentially damage cellular components, leading to death. Nature has evolved to combat this stress by developing antioxidants that enable removal of these oxidative species. These antioxidants can be broadly classified as enzymatic (superoxide dismutase, catalase) or non-enzymatic antioxidants (bilirubin, glutathione). While they are directly involved in eliminating reactive species, cells have evolved other mechanisms that can indirectly reduce or inhibit generation of reactive species, such as heme oxygenase-1, ferritin, ceruloplasmin and glutathione transferase.

A beneficial role for antioxidants has been extensively investigated in both cell culture and animal models of injury and disease (reviewed in [354–376]. However, translation of these studies to the clinical setting has yielded confounding results (reviewed in [360,377–385]). In summary, it appears that supplementation of exogenous antioxidants in several clinical trials had no effect or led to an increase in mortality. Several explanations have been suggested to explain these findings. One school of thought believes that cells employ homeostatic mechanisms to restrict the total allowable antioxidant activity. Therefore, supplying exogenous antioxidants may decrease the rate of synthesis or uptake of antioxidants, so that the total antioxidant potential remains unaltered. Yet another explanation could be simply that the amount of antioxidant is insufficient and is not targeted to the site of excessive reactive species generation. In addition, given the importance of these molecules in signaling and other cellular activities, it may be plausible that their complete removal may lead to altered cellular mechanisms and hence worse outcomes. Furthermore, the relative specificity and efficiency of exogenous antioxidants to reduce each of these reactive species may be different. More importantly, the oxidants responsible for injury must be evaluated. For instance, in a model of cisplatin-mediated renal epithelial injury, overexpression of MnSOD was protective whereas, catalase overexpression was ineffective [386]. This supports the notion that injury may be mediated through different oxidative and nitrosative species and future therapies must be targeted based on the detrimental species generated. Therefore, despite the advances made in deciphering the molecular mechanisms that are regulated by oxidants and antioxidants, translation of these pathways to benefit mankind is still in its infancy and more studies are warranted.

## 11. Concluding Remarks

The field of free radicals has evolved over the past few decades and has significantly contributed to understanding normal physiology and pathophysiology. Research has provided irrefutable evidence that reactive oxygen and nitrogen species are important mediators of cellular response to stress and they function through several mechanisms including, modulation of autophagy, mitochondrial network, signaling and apoptosis. However, high levels of certain reactive species can contribute to cell injury and progression of diseases. These studies granted opportunities for implementing the use of antioxidants in clinical trials, only some of which provided promising results. Therefore, there is an urgent need to comprehensively assess the amounts of different species generated during injury and their relative role in the pathogenesis of disease. Targeting the detrimental reactive species through antioxidant therapy would perhaps yield better outcomes in the clinical trials. In this review, we provided a brief discussion of some of the major pathways that are regulated by ROS and RNS. This review has focused on the complexities and dual roles of these species in cellular activities that affect health and disease. This review will not only provide an understanding of the intricate underlying mechanisms of the reactive species, but will also enable opportunities for the design and development of effective novel therapeutic strategies.
